# A cross-linguistic, sound symbolic relationship between labial consonants, voiced plosives, and Pokémon friendship

**DOI:** 10.3389/fpsyg.2023.1113143

**Published:** 2023-02-24

**Authors:** Alexander Kilpatrick, Aleksandra Ćwiek, Eleanor Lewis, Shigeto Kawahara

**Affiliations:** ^1^Nagoya University of Commerce and Business, Nagoya, Japan; ^2^Leibniz Center for General Linguistics (ZAS), Berlin, Germany; ^3^Independent Researcher, Melbourne, VIC, Australia; ^4^Keio University, Minato, Japan

**Keywords:** cross linguistic analysis, Pokémon, sound symbolism, Poisson regression analysis, iconicity

## Abstract

**Introduction:**

This paper presents a cross-linguistic study of sound symbolism, analysing a six-language corpus of all Pokémon names available as of January 2022. It tests the effects of labial consonants and voiced plosives on a Pokémon attribute known as *friendship*. Friendship is a mechanic in the core series of Pokémon video games that arguably reflects how friendly each Pokémon is.

**Method:**

Poisson regression is used to examine the relationship between the friendship mechanic and the number of times /p/, /b/, /d/, /m/, /g/, and /w/ occur in the names of English, Japanese, Korean, Chinese, German, and French Pokémon.

**Results:**

Bilabial plosives, /p/ and /b/, typically represent high friendship values in Pokémon names while /m/, /d/, and /g/ typically represent low friendship values. No association is found for /w/ in any language.

**Discussion:**

Many of the previously known cases of cross-linguistic sound symbolic patterns can be explained by the relationship between how sounds in words are articulated and the physical qualities of the referents. This study, however, builds upon the underexplored relationship between sound symbolism and abstract qualities.

## 1. Introduction

There has been a growing interest in the exploration of sound symbolism in natural language. Studies that explore this subject typically find that the physical properties of entities are reflected in the speech sounds of their names (Westermann, 1937; Berlin, 1992, 2006; Shinohara and Kawahara, 2010; Sidhu and Pexman, 2018; Davis et al., 2019). These findings challenge one of the principles of the linguistic sign put forth by Saussure (1916) which assumes that the relationship between the form of the linguistic sign and its meaning is arbitrary. However, Sapir's (1929) seminal experimental study on sound symbolism showed that English speakers tend to associate the open vowel /a/ with larger objects and the closed vowel /i/ with smaller objects. Arguably, Sapir’s findings reflect a physical, sensory-based relationship between the object and the sound; open (i.e., lower) vowels reflect largeness because the oral aperture must be larger to articulate them, in comparison to closed (i.e., higher) vowels (Kawahara, 2021).

The current paper contributes to the growing literature on the relationships between abstract qualities and sound symbolism. It examines sound symbolic relationships in the names of North American English (hereafter: English), Japanese, South Korean (hereafter: Korean), Mandarin Chinese (hereafter: Chinese), Standard German (hereafter: German), and European French (hereafter: French) Pokémon. More specifically, it examines a Pokémon attribute known as *friendship* in English and *natsuki-do* [degree of attachment] in Japanese, demonstrating a systematic relationship between this video game metric and the sounds that make up 5,388 video game character names.

### 1.1. Sound symbolism

Sound symbolism, the correspondence between sounds and meaning, has been a popular scholarly area of enquiry since the early 20th century (e.g., Wells, 1919; Sapir, 1929; Newman, 1933) and quantitative research has revealed many sound symbolic patterns, several of which hold cross-linguistically. For example, voiced obstruents have been found to be judged as suitable for the names of larger or heavier objects (Newman, 1933). This pattern has been observed in English (Newman, 1933), Japanese (Hamano, 1986; Shinohara and Kawahara, 2010; Kawahara and Shinohara, 2012), and Chinese (Shinohara and Kawahara, 2010). Alongside size, shape has also been found to be reflected sound symbolically. Köhler (1929) was the first to notice that angular objects tend to be associated with words like *takete* while round objects tend to be associated with words like *maluma,* the effect subsequently replicated with *kiki* and *bouba*, respectively (Ramachandran and Hubbard, 2001). This cross-modal correspondence between sound and shape is known to hold across different languages (Bremner et al., 2013; Chen et al., 2016; Ćwiek et al., 2022, but see Rogers and Ross, 1975; Styles and Gawne, 2017). It is yet unclear, whether consonants or vowels play the main role in the kiki/bouba effect, or whether it relies on their additive symbolic relation (Nielsen and Rendall, 2013; Fort et al., 2015; McCormick et al., 2015). Most recently, Fort and Schwartz (2022) showed that the effect lies in the physical properties of shapes that result in acoustic patterns corresponding to the respective nonce words. Some scholars have also argued that this particular shape–sound relationship may be relevant for the acquisition and development of language (Maurer et al., 2006; Imai and Kita, 2014; Ćwiek et al., 2022).

Several theories have been presented to explain the relationship between sounds and physical properties. Regarding the size–sound relationship, Ohala (1994) proposed the underlying mechanism to be the “frequency code”. In the natural environment, animals that are larger naturally produce a lower fundamental frequency (F0). In turn, lower F0 is perceived as a signal of largeness, and therefore threat and dominance, while higher F0 is perceived as a signal of smallness that stands for the lack of threat and friendliness. It has been shown that some animals use this correspondence as a deceiving mechanism in order to signal hostility (Morton, 1994; Bee, 2000). Ohala (1994, p. 335) also refers to the second formant frequency (F2) as a possible cause for this effect in sound symbolism. F2 frequency has been found to iconically express deictic relations (Johansson and Zlatev, 1970; Ultan, 1978; Woodworth, 1991; Traunmüller, 1994) such that a proximal reference is conveyed by vowels with higher F2, such as [i], and distal by vowels with low F2, such as [a] and [o]. Johansson and Zlatev (1970), after Traunmüller (1994), propose that the proximal–distal and small–large oppositions are based on the iconic similarity between mappings; i.e., a small size corresponds to a small distance, and a large size to a large distance.

Thus, the correspondences described above rely on iconicity – a relationship between the linguistic form and meaning (e.g., Perniss and Vigliocco, 2014). They are not restricted to speech sounds but extend to other linguistic domains, such as prosody. It has been shown that listeners infer information regarding size (Shintel et al., 2014) or movement (Shintel and Nusbaum, 2007) of an entity from prosodic information. Higher pitch represents smaller entities, and faster speech rate represents entities in faster motion. These mechanisms are also conveyed in speech production. It has been shown that speakers reliably use higher pitch to convey “smaller” domains (Nygaard et al., 2009; Perlman et al., 2015), or exhibit higher speech rate when talking about fast events (Shintel et al., 2006; Perlman, 2010; Perlman et al., 2015). Some aspects of iconic prosody can be transferred to the written domain. Fuchs et al. (2019) investigated adjective antonym pairs, such as short/long, tiny/huge, and fast/slow, in an English blogger corpus. They found that the adjectives belonging to an iconically “larger” domain exhibit letter replications more often than the adjectives that belong to the “smaller” domain. Therefore, it seems that, iconically, the amount of the meaning can be expressed with the amount of sounds.

Cross-linguistic iconic coding principles may be natural tendencies to express meanings with sounds that correspond to the physical nature of entities. Human languages are produced by a shared physiology, so it makes sense that we find cross-linguistic patterns whereby large objects are represented by large (or more) sounds and round objects are represented by sounds that are produced with lip rounding gestures. Regarding the expression of size, Winter and Perlman (2021) found that, while general vocabulary of English may not appear to show sound-symbolic patterns in its entirety, its specific subset, namely size adjectives, does so. In English size adjectives, /ɪ/, /i/, /ɑ/, and /t/ are especially suggestive of size – /ɑ/ is more likely to occur in large adjectives, whereas the phonemes /ɪ/, /i/, and /t/ are more likely to occur in small adjectives. For example, words like *tiny*, *itsy-bitsy*, and *large* adhere to this rule, although words like *big* and *small* are exceptions. In a study regarding the expression of shape, Sidhu et al. (2021) examined 1,757 English singular object nouns in the context of the maluma/takete effect (also known as kiki/bouba, see above). They found that the English lexicon carries sound-symbolic information such that phonemes associated with roundness are more likely to be found in names for round objects, and conversely “spiky” phonemes are more common in names for spiky objects (Fort and Schwartz, 2022).

The correspondences between sounds and certain properties that involve a physical representation, such as size or shape, are perhaps the most apparent to us at the first sight. However, those immediate analogies can extend to relationships between speech sounds and more abstract qualities such as politeness (Winter et al., 2021; Brown et al., 2022), humour (Westbury and Hollis, 2019; Dingemanse and Thompson, 2020), or rudeness (Aryani et al., 2018). For example, studies have shown that human languages signal emotion *via* sound symbolism. Adelman et al. (2018) examined word-initial phonemes in English, Spanish, Dutch, German, and Polish and found that across languages, the first phoneme in each word is a significant predictor of emotional valence. They also found that phonemes that were uttered faster tended to convey danger, proposing that emotional sound symbolism is an evolutionary adaptation in humans, akin to alarm calls found in animal communication systems. Perhaps more striking examples of abstract qualities and sound symbolism come from marketing research, where sound symbolism is used to explore possibilities for enhancing the branding strategy (Klink, 2000, 2001, 2003; Pathak et al., 2020, 2022 to name a few). Klink (2000) conducted a comprehensive study comparing word pairs with contrasting segments and their effect on various physical and abstract qualities. The result suggests that native speakers of American English perceive front vowels not only as smaller or faster, but, e.g., also as more feminine, friendlier, or prettier. Except for femininity, these findings echo previous results by Fónagy (1963). As mentioned before, all those abstract qualities seem to be extensions of the basic principles, the immediate physical relationships. These relationships suggest that friendliness is connected with, e.g., high-frequency sounds (Morton, 1977; Ohala, 1994).

Bridging the way towards the main motivation of the current study, previous studies on sound symbolism suggest that in Japanese, voiced obstruents tend to be associated with negative images (Hamano, 1986), while bilabial consonants are often associated with infancy (Kumagai and Kawahara, 2017; Kumagai, 2020). The latter finding is likely grounded in speech development. Babies use their lips to suck milk, and as such, they are a highly relevant organ for them; as a result, labials are among the very first consonants they produce (MacNeilage and Davis, 2000). This finding motivated a study by Uno et al. (2020) which explored sound symbolism in names of villainous and non-villainous characters. They used a corpus of the names of English Disney characters and the names of Japanese Pokémon. Pokémon have associated “types” and Uno et al. used “fairy type” as a proxy for non-villainous and “dark type” (in Japanese *aku-taipu* [evil type]) as a proxy for villainous. In a similar analysis as is found in the present study, they found that across both English and Japanese, voiced obstruents were associated with villainous names, and bilabial consonants were associated with non-villainous names. Motivated by the findings of these previous studies (Hamano, 1986; Kumagai and Kawahara, 2017; Kumagai, 2020; Uno et al., 2020, and others), the current study examines the sound symbolic properties of labial consonants and voiced obstruents. We take the immediate physical relationship and explore the extension of this relationship onto the abstract quality of friendliness in the names of Pokémon.

### 1.2. Pokémonastics

Pokémon—also known as *Pocket Monsters* in Japan—is a Japanese media franchise centered on fictional monsters called Pokémon. Human characters in the franchise—known as trainers—catch and train Pokémon to battle with other Pokémon trainers for sport. The franchise began as a video game which soon developed into a mixed media empire. In the video game world, Pokémon have attributes that are expressed as numerical values. Pokémonastics is a relatively new field of research (Kawahara et al., 2018 et seq.) that examines sound symbolic relationships between the names of Pokémon and these attributes.

Pokémonastic studies have some advantages over natural language studies and may uncover interesting cross-linguistic, sound symbolic relationships that would otherwise remain unobserved. Pokémon has been translated into many different languages, and while names change, Pokémon imagery and attributes remain stable. For example, the Pokémon known as *Bulbasaur* in English, is known as *Miàowāzhǒngzǐ* in Chinese, and while they have different names, all other attributes and images remain the same. Cross-linguistic Pokémonastic studies therefore do not have to deal with issues pertaining to lexical semantics or polysemy that natural language studies may encounter (see Kawahara and Breiss (2021) for detailed discussion on this point).

Initially, Pokémonastic studies focused on Japanese Pokémon names and found that features such as the number of voiced obstruents and name length reflected Pokémon attributes such as increased size and strength parameters (Kawahara et al., 2018). These studies have since been expanded to examine Pokémon names cross-linguistically to determine whether the same patterns exist across languages. In one such study, Shih et al. (2018) found that increased name length represented increased size, power, and evolution level not only in Japanese but also in English Pokémon. As Pokémon battle, they gain experience, and once they have gained enough experience, their level, and attributes increase. Many Pokémon have the option to evolve once they have attained a certain level which typically results in increased size and combat ability. Given that size, power, and evolution level are related, this finding can arguably be attributed to the *iconicity of quantity* (Haiman, 1980)—whereby larger entities are expressed by longer words as both strength and size (Shih et al., 2018) and evolution level can be symbolically expressed in terms of the length of Pokémon names (Kawahara and Moore, 2021).

An interesting hypothesis that has arisen from Pokémonastic studies is that elements of sound symbolism in human languages may reflect attributes that are crucial to evolutionary survival (Shih et al., 2018; Uno et al., 2020). Pokémon attributes that reflect survivability—such as size and combat parameters—seem to be robustly reflected sound symbolically in Pokémon names while parameters that have little effect on survivability—such as gender distribution—are not. While in the real world, gender might predict combativeness, size, and physical strength, the gender mechanic in the video games has little in-game effect other than determining suitable partners in Pokémon breeding (Bulbagarden, 2004). Although not explicitly stated by Shih et al., this suggests that elements of sound symbolism relating to survivability can be innate (Perniss et al., 2010), presumably because they are evolutionarily beneficial. This proposal raises the possibility that human language evolved rather as a multimodal than a purely gestural system. Arguments for and against this proposal are presented in Kendon (2017) and Tomasello (2008) respectively (see also Perlman, 2017; Fröhlich et al., 2019; Ćwiek et al., 2021). Indeed, several recent studies point to the importance of multimodality in the emergence of language (Macuch Silva et al., 2020; Nölle et al., 2020). This is one of the reasons why it is important to explore sound symbolic patterns that reflect abstract qualities because cross-linguistic patterns that reflect physicality can be explained by natural and iconic relationships between sounds and objects, but when a quality has no physical representation, this explanation cannot hold (Lupyan and Winter, 2018). In order to contribute to addressing this general question, the present study seeks to test sound symbolic relationships pertaining to Pokémon friendship. This is with a caveat in mind that whether or not friendship is an abstract quality is debatable given that friendship in the Pokémon universe may be interpreted as a lack of threat or a lack of dominance which are conceptualized as concrete qualities in the frequency code hypothesis (Ohala, 1994).

The present study examines a previously unexplored feature of Pokémon in Pokémonastics studies known as friendship. Friendship was first introduced in Pokémon Yellow, an enhanced version of the first generation of the core series of Pokémon video games, where it was only applied to the player’s starter Pokémon. From the second generation onwards, all Pokémon had friendship as an attribute; however, in the second generation of the core series of video games, friendship was known as *loyalty*. Friendship has also been known as *tame* in Pokémon Stadium 2, *friendliness* in Pokémon XD: Gale of Darkness, and is often referred to as *happiness* by the gaming community. A second attribute known as *affection* in English or *nakayoshi-do* “degree of closeness” in Japanese was introduced in the sixth generation of the core video games, but this attribute was integrated into friendship in the eighth generation. The friendship level is expressed as a value ranging from 0–255. Each Pokémon has a default level of friendship, and in the following, we refer to this value as *base friendship*. Base friendship is the friendship value that each Pokémon has when it is first encountered by the player. Prior to generation 8 of the core series of video games, most Pokémon had a base friendship of 70; however, this was reduced to 50 for most Pokémon when affection was integrated into friendship. The present study uses the most recent base friendship values obtained from Bulbagarden (2004).

Applying real-world meaning to friendship can be difficult because friendship has many different in-game effects. Friendship affects the base power of certain attacks, whether a Pokémon may evolve, non-player character interactions, and access to certain areas in the game world. If a trainer treats their Pokémon well (such as healing it during battle) the friendship value increases; on the other hand, if they treat their Pokémon poorly (such as making it battle until it loses consciousness), the value decreases. Pokémon can be traded between players and when a Pokémon is traded, all its attributes remain the same except for its friendship attribute which reverts to base, i.e., default friendship mentioned above, unless it is being traded back to a previous owner. Base friendship may therefore be considered a metric that relativises how naturally friendly each Pokémon is because it reflects how quickly Pokémon become attached to their trainers. Alternately, base friendship may be considered a reflection of how naturally happy each Pokémon is because those events that affect friendship would also affect mood. Yet another interpretation is that base friendship reflects how tame each Pokémon is because friendship affects how well Pokémon follow commands by raising the base power of certain moves. A common thread that runs through each of these interpretations is that base friendship reflects how threatening each Pokémon is to the player character because entities that are friendly, happy, and tame are less threatening than those that are not. These reasons taken together motivate using the base friendship metric as a baseline to study the sound-symbolic expression of natural friendliness of Pokémon.

### 1.3. Specific hypotheses tested

The present study uses phoneme frequency in Pokémon names as a predictor of base friendship. It tests the following hypotheses:

1. Labial consonants (/p/, /m/, and /w/^1^) will occur more frequently in the names of Pokémon with high base friendship values.
2. Voiced obstruents (/d/ and /g/) will occur more frequently in the names of Pokémon with low base friendship values.
3. Given that /b/ is both a bilabial consonant and a voiced obstruent, we make no prediction as to its relationship with base friendship.

The first hypothesis is motivated by the findings of studies that show a relationship between labial sounds and infancy. Labials are among the very first sounds produced by infants (MacNeilage and Davis, 2000), which may motivate our association of those sounds with baby-like features (Kumagai and Kawahara, 2017) and images of softness and cuteness (Kumagai, 2020). Pokémon with high friendship values—much like infants—are non-threatening, and labial sounds may be a way of communicating this.

The second hypothesis stems from the findings of Uno et al. (2020), which shows that voiced obstruents were predictors to villainy. Numerous studies have also shown that voiced obstruents have a systematic relationship with size, whereby objects with voiced obstruents in their name are more likely to be larger (Newman, 1933; Hamano, 1986; Shinohara and Kawahara, 2010; Kawahara and Shinohara, 2012). Villainous and large objects are more threatening than non-villainous and small objects so much like the way that bilabial consonants may communicate that an object is non-threatening, voiced obstruents may communicate danger. The voiced bilabial plosive/b/, is a special case because it is both a labial consonant and a voiced obstruent, so it is unclear whether it will be associated with high/low base friendship.

## 2. Materials and methods

All data and code for this study are available under the following OSF repository: https://tinyurl.com/59xhxknr.

The data for this study was extracted from the website Bulbagarden (2004). Bulbapedia provides names for Pokémon in many languages; however, the present study only examines those languages that have been incorporated into the main content spaces (mainspaced, in the parlance of the website) as of January 2022. Mainspacing is used as quality control by the editors of the website; once an article has been mainspaced, it is considered complete. Only English, Japanese, Chinese, Korean, German, and French Pokémon tables have been mainspaced.

The following analysis is conducted on a phoneme count of the labial consonants, /p/, /b/, /m/, and /w/, and the voiced plosives, /b/, /d/, and /g/. The phoneme count was conducted by individuals with training in phonology and/or phonetics who were either native speakers or knowledgeable of the phonology of the languages in question. None of the individuals who conducted the phoneme counts consider themselves familiar with all generations of Pokémon, so the analyses were not based on existing knowledge about Pokémon. For Japanese, Korean, and Chinese, the phoneme count was based on an algorithm designed to extract phoneme counts from Katakana, Hangul, and Pinyin. Japanese, Korean, and Chinese were treated differently to English, German, and French because Katakana, Hangul, and Pinyin scripts are reasonably phonetic. The accuracy of this initial analysis was then assessed by native speakers of each respective language and adjustments were made where appropriate. For English, German, and French, phoneme counts were based on a combination of an acoustic analysis, using audio samples from the animated television program, as well as graphemic and morphemic analyses. When phonemic identity was clear and unambiguous based on either a graphemic or a morphemic analysis, audio samples were not sought. For example, an audio sample was not sought in the case of the English Pokémon *Blastoise* because it is clear from both its spelling and its morphemes (blast + tor[toise]) that of the target phones, there is only a single occurrence of /b/. On the other hand, whether there is a /g/ in the name of the English Pokémon *Yungoos* is ambiguous. In instances like this, we sought audio samples from the animated television program to determine phonemic identity. On occasion, audio samples from the animated television program could not be sourced. This occurred twice in the English data set with the Pokémon named *Regialeki* and *Regidrago*, where it is unclear whether <g> represents the voiced velar plosive or the voiced postalveolar affricate. In these cases, we examined the linguistic behaviour of unofficial content creators whose content suggested that they were native Pokémon speakers.

In the case of German, conversion from orthography to the phonological realization was implemented. This process implemented a set of rules, such as final devoicing of free morphemes, but it also encompassed some rules for the realization of <s> in German, as /s/, /z/, or /ʃ/. However, because both the realization of <s> in cases of novel names and, more importantly, varying assimilation that influences voicing could not be relied upon, all names were checked by the second author who is a phonetician proficient in German. The manual control resulted in identifying problematic cases, especially involving the assimilation issues. For example, in Taubsi “Pidgeotto”, <b> is devoiced as a result of regressive assimilation to /s/. There were some cases in which the automatic conversion recognized the segment as devoiced, even if it remained voiced, e.g., in Sengo “Zangoose”, <g> pertains the voicing. In many cases, similarly to English, we relied on audio samples from the Pokémon community to seek for the most likely pronunciation. Since there is a lot of variation in German pronunciation, we sought multiple samples for the same name. This procedure allowed us to correct the automatic conversion in names such as Gelatini “Vanillite,” Gelatroppo “Vanillish,” and Gelatwino “Vanilluxe” regarding the initial <g> that is based on Italian pronunciation as an affricate [d͡ʒ], which is actually devoiced in German and realized as [t͡ʃ].

In the case of French, it was difficult to find official recordings of many of the Pokémon names, so the services of a native French professional voice-over artist with considerable experience with the Pokémon franchise was engaged. A recording of the speaker was made, and the analysis was conducted by the third author, a non-native French speaking phonetician. This process was only done in the case of French because it was difficult to find official audio samples from which to analyse.

In all languages except for Chinese and Korean, all target phones were counted. In Chinese (Duanmu, 2007, p. 24) and Korean (Sohn, 2019), there is no phonological opposition between voiced and voiceless plosives. Therefore, there is no data for/b/, /d/, and /g/ in these languages. Both languages do have phonological opposition between aspirated and unaspirated plosives, and on the advice of the volunteers who assessed the accuracy of the Chinese and Korean data, counts for aspirated and unaspirated /p/ were conducted separate to the initial /p/ count. Korean plosives are often voiced when they occur intervocalically (Sohn, 2019), and on the advice of the Korean volunteer, a count of intervocalic plosives was conducted. Aspirated and unaspirated /p/ in Chinese and Korean, and intervocalic /p/, /t/, and /k/ in Korean are not included in the cross-linguistic models and are analysed separately. Japanese and Korean have phonemically contrastive gemination on plosives; however, geminates were treated as single instances of the same sound because they have one place of articulation and one closure movement (i.e., they are simply long consonants, instead of consonants that involve double articulation).

Since the analysis was conducted, two new Pokémon video games have been released which both include new canonical Pokémon; Pokémon Legends Arceus and Pokémon Scarlet/Violet (Generation 9 of the core series). These new Pokémon are not included in the present study. Therefore, the present study examines the names of 898 Pokémon spanning generations 1 through 8 (1996–2021) in six languages resulting in 5,388 tokens.

In the following sections, we examine the relationship between voiced plosives, labial consonants, and base friendship. Unlike in previous Pokémonastic studies, Poisson regression is used to predict the occurrence of certain sounds based on base friendship, because while base friendship is represented numerically, it is not a continuous variable (Winter and Bürkner, 2021). Table 1 reports the distribution of Pokémon per level of base friendship.

**Table 1:** Distribution of Pokémon per level of base friendship.

Base friendship	Number of Pokémon
0	48
35	73
50	744
70	2
90	5
100	15
140	11

It is important to note that a certain amount of name borrowing occurs between languages. For example, the Pokémon known as *Pikachu* in English (base friendship = 50), is *P′ik’ach’yu* in Korean McCune-Reischauer romanization, *Píkǎqiū* in Chinese Pinyin, and *Pikachu* in Japanese, German, and French. While Pikachu is a special case because it is a mascot for the franchise, the reader should be made aware that borrowing does occur between languages. As an illustration of borrowing across languages, Table 2 presents a list of the names of all starter Pokémon from the 8 generations of core games in their pre-evolution form. In the present study, no Pokémon names have been excluded from the analysis due to being borrowed from another language, because exclusion would need to be based on an arbitrary perception of similarity. This can be raised as a concern in a study that draws conclusions based on cross-linguistic patterns. However, there is enough cross-linguistic variability to suggest that when a name is borrowed from one language to another, it has been deemed appropriate for said language.

**Table 2:** List of each pre-evolution starter Pokémon from all 8 generations of the core Pokémon video games.

English	Japanese	Korean	Chinese	German	French
Bulbasaur	Fushigidane	Isanghaessi	Miàowāzhǒngzǐ	Bisasam	Bulbizarre
Charmander	Hitokage	P’airi	Xiǎohuǒlóng	Glumanda	Salamèche
Squirtle	Zenigame	Kkobugi	Jiéníguī	Schiggy	Carapuce
Chikorita	Chikorīta	Ch’ik’orit’a	Júcǎoyè	Endivie	Germignon
Cyndaquil	Hinoarashi	Pŭk’ein	Huǒqiúshǔ	Feurigel	Héricendre
Totodile	Waninoko	Riak’o	Xiǎojù’è	Karnimani	Kaiminus
Treecko	Kimori	Namujigi	Mùshǒugōng	Geckarbor	Arcko
Torchic	Achamo	Ach’amo	Huǒzhìjī	Flemmli	Poussifeu
Mudkip	Mizugorō	Multchangi	Shuǐyuèyú	Hydropi	Gobou
Turtwig	Naetoru	Mobugi	Cǎomiáoguī	Chelast	Tortipouss
Chimchar	Hikozaru	Pulkkotsungi	Xiǎohuǒyànhóu	Panflam	Ouisticram
Piplup	Potchama	P’aengdori	Bōjiāmàn	Plinfa	Tiplouf
Snivy	Tsutāja	Churibiyan	Téngténgshé	Serpifeu	Vipélierre
Tepig	Pokabu	Ttukkuri	Nuǎnnuǎnzhū	Floink	Gruikui
Oshawott	Mijumaru	Sudaengi	Shuǐshuǐtà	Ottaro	Moustillon
Chespin	Harimaron	Toch’imaron	Hālìlì	Igamaro	Marisson
Fennekin	Fokko	P’uhokko	Huǒhúlí	Fynx	Feunnec
Froakie	Keromatsu	Kaegumarŭ	Guāguāpàowā	Froxy	Grenousse
Rowlet	Mokurō	Namolppaemi	Mùmùxiāo	Bauz	Brindibou
Litten	Nyabī	Nyaobul	Huǒbānmiāo	Flamiau	Flamiaou
Popplio	Ashimari	Nurigong	Qiúqiúhǎishī	Robball	Otaquin
Grookey	Sarunori	Hŭngnasung	Qiāoyīnhóu	Chimpep	Ouistempo
Scorbunny	Hibanī	Yŏmbŏni	Yántù’er	Hopplo	Flambino
Sobble	Messon	Ulmŏgi	Lèiyǎnxī	Memmeon	Larméléon

Japanese names are written in Hepburn romanization, Korean names are written in McCune–Reischauer romanization, and Chinese names are written in Pinyin.

### 2.1. Statistical analysis

All analyses were performed in R version 4.2.2 (R Core Team, 2013). For a full list of packages and versions, interested readers are referred to the annotated script in the following repository: https://tinyurl.com/59xhxknr. The central hypothesis of the present study is that Pokémon names carry sound-symbolic information pertaining to the base friendship metric. In other words, when a listener hears a Pokémon name, they can make a better than chance assessment as to its friendship. To statistically test this hypothesis, we use a series of Poisson regression models with base friendship as the dependent variable and the sounds in their names as the predictor variables. Poisson regression models are a type of generalized linear model that are used to model non-continuous data. Since our dependent variable is not continuous, Poisson regression was deemed suitable for this task (for a clear exposition for this statistical analysis, see Winter (2019): Ch 13 as well as Winter and Bürkner (2021)). As is shown in Table 1, the distribution of Pokémon per level of base friendship is highly skewed, with most Pokémon having a base friendship value of 50.

We also explore the potential that base-friendship reflects size and combat ability, i.e., that bigger, stronger Pokémon are unfriendly, and that the patterns observed might model better to other metrics. To test this, we use a multivariate regression analysis examining the relationship between base friendship and combat and size attributes. Lastly, we use a series of multivariate Poisson regression analyses examining base friendship, size, and combat variables and the frequency of each phoneme to tease apart base friendship from these other metrics.

## 3. Results

A descriptive analysis shows that there is a relationship between base friendship and the frequency of the six phonemes, whereby /p/ occurs most frequently in Pokémon with a base friendship of 140, while /g/ and /d/ occur most frequently in Pokémon with a base friendship of 0. Table 3 reports the average number of times that each phoneme occurs in each Pokémon name distributed by base friendship level.

**Table 3:** Average number of times that each phoneme occurs in Pokémon names by base friendship level.

Base friendship	0	35	50	70	90	100	140
/p/	0.09	0.16	0.24	0.33	0.03	0.27	0.48
/b/	0.06	0.15	0.19	0.25	0.15	0.18	0.07
/d/	0.38	0.23	0.28	0.33	0.07	0.24	0.20
/m/	0.21	0.26	0.20	0.25	0.30	0.08	0.09
/g/	0.32	0.21	0.19	0.13	0.05	0.07	0.02
/w/	0.03	0.04	0.06	NA	NA	0.06	0.02

A generalized linear mixed-effects model was constructed with language included as a random variable to test the effect of phoneme frequency on base friendship (results reported in Table 4). These results show a significant effect for all phonemes other than /w/ (*p* < 0.001 in all cases) on base friendship where bilabial plosives (/p/ and /b/) are associated with high base friendship while /d/, /m/, and /g/ are associated with low base friendship.

**Table 4:** Results of the generalized linear mixed-effects model applied to all samples testing the effect of phoneme count on base friendship.

Preds.	*R* ^2^	*z*-value	*p*
/p/	0.044	15.74	<0.001
/b/	0.006	4.78	<0.001
/d/	0.009	−5.73	<0.001
/m/	0.011	−7.55	<0.001
/g/	0.05	−13.24	<0.001
/w/	0	0.87	0.387

A second generalized linear mixed-effects model was constructed with language included as a random variable. This model was constructed to try to negate the influence of cross-linguistic borrowing that may exist in the previous model. To achieve this, any Pokémon whose phoneme count matched that of the same Pokémon in a different language was excluded from the analysis. For example, Pikachu, whose name consists of a single instance of /p/ in all languages, was only counted once in the dataset. This method also excluded Pokémon whose names were clearly not the result of borrowing. For example, the Pokémon known as ズルズキン *zuruzukin* in Japanese and *scrafty* in English are clearly not related. However, only one of these samples was included in the analysis because both names contain zero instances of all the examined phonemes, and therefore they have the same phoneme count. An alternate method for excluding names was considered whereby each name sextuplet would be manually examined and a judgement would be made as to whether there was evidence of borrowing. This method was deemed to be inappropriate given the arbitrary nature of the judgment call and for reasons addressed in the conclusion. In total, 2,124 samples were excluded from the previous model, resulting in 3,264 samples. The results of the second generalized linear mixed-effects model are presented in Table 5. The model revealed a significant effect for phonemes /p/, /d/, and /g/ of phoneme count on base friendship where /p/ is associated with high base friendship while /d/ and /g/ are associated with low base friendship.

**Table 5:** Results of the second generalized linear mixed-effects model applied to all samples with unique phoneme counts.

Preds.	*R* ^2^	*z*-value	*p*
/p/	0.017	7.53	< 0.001
/b/	0.001	1.86	0.063
/d/	0.002	−2.08	0.037
/m/	0	−0.22	0.823
/g/	0.015	−6.39	< 0.001
/w/	0	−0.5	0.620

Following the cross-linguistic models, a series of generalized linear models were constructed to test the effects of phoneme count on base friendship in languages independently. This analysis was conducted on the entire dataset because it is not always clear which is the source language in borrowing. Those patterns observed in the generalized linear mixed-effects models generally held true when individual languages were examined. Bilabial plosives /p/ and /b/ were found to be significant predictors of high base friendship in all cases except for /b/ in French (see Table 6). The voiced non-bilabial plosives /d/ and /g/ were significant predictors of low base friendship in all cases other than the case of /d/ in English and French. Interestingly, the bilabial nasal /m/ was a significant predictor of low base friendship in all languages despite not achieving significance in the generalized linear mixed-effects model designed to limit the influence of name borrowing. /w/ was not found to be a significant predictor of base friendship in any language.

**Table 6:** Z-scores for the regression analyses for the six phonemes across the six languages.

	English	Korean	Chinese	Japanese	German	French
/p/	4.15^***^	7.6^***^	5.9^***^	10.86^***^	7.04^***^	4.17^***^
/b/	2.91^**^	NA	NA	1.65	4.83^***^	0.56
/d/	−1.28	NA	NA	−4.57^***^	−4.77^***^	−1.08
/m/	−1.98^*^	−4.35^***^	−2.67^**^	−3.08^**^	−4.28^***^	−2.09^*^
/g/	−7.36^***^	NA	NA	−5.07^***^	−9.37^***^	−5.06^***^
/w/	−0.8	0.66	0.07	0.57	0.4	1.6

Asterisks reflect significance where ^*^p < 0.05, ^**^p < 0.01, and ^***^p < 0.001.

These findings mirror those findings in earlier studies that have shown that voiced obstruents are related to increased referent size (e.g., Newman, 1933; Hamano, 1986) while bilabial consonants are related to cuteness in English and Japanese (Uno et al., 2020) and possibly other languages, so that what we are finding is possibly a relationship between size (or power) and sound rather than friendliness. To examine a potential relationship between base friendship, size (height and weight), and the sum of the combat aptitude parameters (attributes known as hit points, attack, defence, special attack, special defence, and speed), we conducted a multivariate regression analysis to explore a potential relationship between combat aptitude, size, and base friendship. As was observed in Kawahara et al. (2018), Pokémon height and weight measures are heavily right-skewed, so we took the natural logarithm of these measures. A significant regression equation was found (*F*(3,5,384) = 140.3, *p* < 0.001) with an *R*^2^ of 0.72. The results of this analysis are presented in Table 7.

**Table 7:** Results of a multivariate regression analysis examining the relationship between base friendship and combat and size attributes.

Metric	*t*-value	*p*
Combat aptitude	7.23	<0.001
Log weight	9.58	<0.001
Log height	0.52	0.604

Combat aptitude and weight explain a significant proportion of variance in base friendship, where Pokémon with low base friendship are significantly heavier and stronger than those with high base friendship. We therefore conducted a series of multivariate Poisson regression analyses examining phoneme frequency as a predictor of base friendship, size, and combat aptitude. Given that /w/ was not found to be significant in any previous model, it was excluded from this analysis. These are reported in Table 8. The results show that base friendship has the strongest effect size with /p/ and /g/ frequency, but not of /b/, /d/, and /m/ frequency.

**Table 8:** Results of a series of multivariate Poisson regression analyses examining base friendship, size, and combat variables and the frequency of each phoneme in all languages.

Phone	/p/		/b/		/d/		/m/		/g/	
Preds.	*z*-value	*p*	*z*-value	*p*	*z*-value	*p*	*z*-value	*p*	*z*-value	*p*
B. Friendship	5.42	< 0.001	1.59	0.112	−0.06	0.951	−4.32	< 0.001	−3.96	<0.001
Combat Total	−2.58	0.010	−3.22	0.001	−0.63	0.529	2.43	0.015	0.96	0.337
Log Height	0.51	0.601	0.01	0.993	3.22	0.001	−5.44	< 0.001	−0.07	0.943
Log Weight	−2.20	0.028	0.15	0.884	0.21	0.833	0.83	0.408	2.64	0.008
	*R*^2^ = 0.25		*R*^2^ = 0.009		*R*^2^ = 0.012		*R*^2^ = 0.019		*R*^2^ = 0.024	

B. Friendship refers to base friendship.

## 4. Discussion

The current study contributes to the body of literature that examines the relationships between sounds and meanings in natural languages. It examines a corpus of over 5,000 Pokémon names from six languages and shows a systematic relationship between the Pokémon attribute known as base friendship and the phonemes that make up the names of Pokémon. Specifically, we hypothesized that labial consonants would occur more frequently in the names of high base friendship Pokémon while low base friendship Pokémon names would contain voiced obstruents.

The findings show varying tendencies for labial phonemes. Bilabial plosives /b/ and /p/ are positively correlated with base friendship, as expected from the results of the previous studies. No effect for /w/ was found in our data. The bilabial nasal /m/, however, was negatively correlated with base friendship in our sample. The last one is a novel result in light of Kumagai (2020) and Kumagai and Kawahara (2017), who found a positive correlation between bilabial segments in general and infancy. In all languages with phonological opposition for voicing on plosives, /d/ and /g/ both showed a tendency to be associated with low base friendship, and this tendency was strongest with/g/ (*z* = −13.24, *p* < 0.001). This suggests that voiced plosives are reflective of increased threat, except in the case of /b/ which was generally associated with low base friendship. Since /b/ is both a voiced plosive and a bilabial consonant, no hypothesis was specified for this phoneme.

Base friendship is arguably an abstract quality, which should have no physical representation. It has, however, been shown that seemingly abstract forms may evoke perceptual, affective, and sensory responses similar to real-world objects. In Lindauer’s (1990) experiment, participants judged the shapes of *takete* and *maluma*, originally conceived by Köhler (1929, 1947), as being the least and the most friendly, respectively, as compared to neutral stimuli. In another experiment, Sidhu et al. (2021) examined how sound symbolism conveys abstract qualities. They investigated the perception of various personality traits in first names. The names were grouped into those that contained sonorants /m/, /n/, and /l/, and those that contained voiceless stops /p/, /t/, and /k/. The results suggest that sonorants and voiceless stops are associated with different personality factors, some of which may be linked to friendliness (Sidhu et al., 2019). Considering Ohala’s (1994) frequency code, infancy is a signal for lack of threat, so it is difficult to explain the relationship between /m/ and low base friendship. Our analysis is the first one that disentangles the connotations of different labial segments and exposes this contradicting effect when considering base friendship rating in Pokémon.

We initially hypothesised that the labial approximant, /w/, would be associated with high base-friendship because, like bilabial consonants, [w] appears to be a sound that infants acquire early in their development. Contrary to our expectation, however, /w/ was associated with neither high nor low base-friendship. Our admittedly post-hoc explanation is as follows. Kumagai and Kawahara (2022) propose that, in Japanese at least, infancy is represented by an abstract phonological feature [+labial] rather than the specific labial gestures of each consonant. In their experiment that explored sound symbolic relationships in the Japanese names of baby diapers, they showed that the voiceless bilabial fricative, /ɸ/, carried the same sound-symbolic associations as other labial consonants, despite this fricative sound being acquired later in development (Ota, 2015). They thus argued that that the relationship between sound symbolic information and age of acquisition/usage frequency in infants is an abstract one, mediated by an abstract phonological feature. One potential explanation for why we observed no sound symbolic association with /w/ in the present study is that perhaps/w/ is not [+labial], or at least not fully [+labial],^2^ because phonetically speaking, /w/ involves both a labial gesture as well as a dorsal gesture; the International Phonetic Alphabet indeed considers [w] as labio-velar. Indeed, whether/w/ is specified as [+labial] or not is at best a contended issue, with /w/ sometimes phonologically behaving like a labial consonant but sometimes now (see, e.g., Nevins and Chitoran, 2008). In short, phonetically speaking, /w/ is not unambiguously labial, as it also involves a narrowing gesture at velar, and perhaps relatedly, /w/ is not unambiguously phonologically [+labial] either. This ambiguous status of /w/ may lie behind the current results.

In the current study we report findings that friendliness may also have a physical representation, as friendly Pokémon tend to be smaller and weaker than their unfriendly counterparts. An attentive reader may nevertheless ask whether the current study tests friendliness explicitly, or whether our results are simply reflecting known size effects in sound symbolism (Newman, 1933; Hamano, 1986; Kawahara and Shinohara, 2012). The Poisson multivariate analysis that was conducted to test this possibility did show that the base friendship model had a stronger effect size than combat, weight, and height models for/p/ and /g/ frequency, but not for other phonemes. Voiced obstruents have been shown to be predictors of size in other Pokémonastic studies (Kawahara et al., 2018). Although it was not found to be a significant predictor in Japanese, Korean, and French, high /b/ frequency trended toward high friendliness in all languages, suggesting that friendliness and size are expressed differently sound symbolically. The relationship between the frequency of the bilabial nasal /m/ and base friendship presents another piece of evidence that base friendship is not simply a reflection of size. /m/ frequency was shown to be a significant predictor of base friendship in all languages, whereby Pokémon with low base friendship generally had a greater occurrence of /m/ in their names. This is a surprising finding given that bilabial consonants (in English and Japanese at least) appear to be associated with cuteness and infancy (Kumagai, 2020). However, it is important to note here that /m/ was not found to be significant in the second generalized linear mixed-effects model which was designed to limit the effects of name borrowing between languages. Therefore, this relationship may be limited to Japanese, or English given that these languages are the most likely candidates as the source for borrowing. Interestingly, /m/ frequency was not a significant predictor of weight, but high /m/ frequency was shown to connote shortness (although this was only shown to be significant in Japanese). Given the negative relationship between base friendship and /m/ frequency, if base friendship is simply a reflection of Pokémon size, then a positive relationship between height and /m/ frequency would be expected. This was clearly not the case.

It is interesting to examine our findings through the lens of the hypothesis promulgated by Shih et al. (2018) that elements of sound symbolism possibly reflect the survivability of Pokémon. Here however, we divert from their assumption that symbolism in human languages communicates the qualities of one’s allies and tentatively put forth our own hypothesis that relates more closely to alarm call theory (Adelman et al., 2018). Pokémon are both allies and enemies in the Pokémon world. In the non-Pokémon world, many non-human primates have elaborate alarm calls that communicate information regarding approaching predators. East African vervet monkeys, for instance, have an alarm call system that differentiates between mammalian, avian, and snake shaped predators (Seyfarth et al., 1980). It is possible, therefore, that elements of sound symbolism in human languages are the result of an alarm call system which early humans used to warn others of approaching danger, as hypothesised by Adelman et al. (2018). In the context of the findings of the present study, perhaps patterns relating to base friendship are the result of an alarm call system that communicated the potential threat of an observed predator. This may explain the apparent sound symbolic relationship between demeanour and size because larger predators would be more likely to engage early humans as prey.

This also may give insight into why the relationship between voiced obstruent frequency and weight appears to have a greater effect size than height in Pokémonastic studies (Kawahara et al., 2018; Shih et al., 2018). In professional combat sports, fighters are separated into weight classes—rather than height classes—because weight is a better predictor of threat. We find this proposition of a sound symbolic pattern that reflects threat more convincing than one that expresses the survivability of one’s allies because of convergent evolution—that under the same environmental pressures, separate species will exhibit similar traits—and the prevalence of alarm call systems in the animal kingdom. That said, we note that the present study does not constitute sufficient evidence to substantiate this hypothesis but discuss it here with the hope that further investigation may yield interesting results.

Ideally, this study would have been conducted on a phonetic analysis, rather than using a count of the target phones. A phonetic analysis would open interesting areas of enquiry into whether sound symbolism is phonetically or phonemically based. Additionally, a phonetic analysis may also provide a more accurate and robust method for excluding borrowed samples. Borrowing can be problematic in a cross-linguistic study because relationships between sounds and meanings that appear to be occurring across languages when they simply occur in the source language. In practice, however, excluding borrowed names in an objective fashion is difficult because it is not always clear when a name is borrowed and when it comes from a similar source. For example, the Pokémon known as *Omanyte* in English resembles a species of extinct marine molluscs known as *ammonites*. *Omanyte* is known as *Omunaito* in Japanese, *Amnait’ŭ* in Korean, *Amonitas* in German, and *Amonita* in French. Given that these names are clearly based on the Latin name for ammonite, it is difficult to ascertain whether these are true borrowings, or whether they are based on the same source. As is shown in Table 2, there is considerable variation between languages in the names of the pre-evolution starter Pokémon. Therefore, we may reasonably determine that even when borrowing does occur, it has been ascertained that the borrowed name—and its sound/meaning associations—is appropriate to the adoptive language.

Finally, an anonymous reviewer noted that those individuals that select the names for Pokémon might also be acquainted with the principles of sound symbolism and therefore might take into consideration these principles in the naming process. Although friendship has not existed in all generations of the video games, this is indeed a possibility, and one that we must consider given that there is little literature on how Pokémon are named. However, this concern does not trivialize our results, we believe, since what we are exploring is ultimately the knowledge of sound symbolism that human beings have. We are unveiling some aspects of such knowledge through the lens of Pokémon names. If the Pokémon designers applied some sound symbolic principles that they have, that is most likely because they wanted to convey the sound symbolic meanings to the customers. What we found, we believe, is thus shared knowledge about how meanings can be expressed in terms of sounds.

## 5. Conclusion

The present study shows a systematic cross-linguistic relationship between frequency of phones and the Pokémon attribute known as base friendship. It shows that /p/ represents high friendliness in all the examined languages, while /g/ is shown to represent unfriendliness in all languages. This is an important finding because friendliness is arguably an abstract quality and can therefore not be explained by a natural relationship between the shape of an object and the shape that the oral cavity conforms to when making the sounds that describe or name said object. The finding that sound symbolic patterns pertaining to friendliness hold cross-linguistically suggests that more is at play in sound symbolism than a natural relationship between shape and sound.

## Data availability statement

Publicly available datasets were analyzed in this study. This data can be found here: https://tinyurl.com/59xhxknr.

## Author contributions

AK: devised and supervised the experiment, conducted the phoneme count for English, created the algorithms for counting phonemes in Chinese, Japanese, and Korean, conducted the statistical analysis, created the first draft of the paper, and editing. AĆ: conducted the phoneme count for German, created the second draft of the paper, and editing. EL: conducted the phoneme count for French and editing. SK: conducted the statistical analysis and editing. All authors contributed to the article and approved the submitted version.

## Funding

This study was supported by Japan Society for the Promotion of Science (Tokyo, JP) grant number: 20K13055. This grant was also used to pay a voice over artist who recorded the French Tokens.

## Conflict of interest

The authors declare that the research was conducted in the absence of any commercial or financial relationships that could be construed as a potential conflict of interest.

## Publisher’s note

All claims expressed in this article are solely those of the authors and do not necessarily represent those of their affiliated organizations, or those of the publisher, the editors and the reviewers. Any product that may be evaluated in this article, or claim that may be made by its manufacturer, is not guaranteed or endorsed by the publisher.

## Footnotes

^1^There is some debate as to whether [w] is phonologically [+labial] or [+dorsal] (see review in Kumagai, 2020).

^2^In the theory of Gradient Symbolic Representation (Smolensky and Goldrick, 2016 et seq), it is possible for a segment to bear a numeric specific activation for a particular phonological feature (e.g., [0.6 labial]).
